# Recent studies on pinene and its biological and pharmacological activities

**DOI:** 10.17179/excli2021-3714

**Published:** 2021-04-22

**Authors:** Byung Bae Park, Ji Young An, Sang Un Park

**Affiliations:** 1 Department of Environment and Forest Resources, Chungnam National University, 99 Daehak-ro, Yuseong-gu, Daejeon 34134, Korea; 2 Department of Crop Science and Department of Smart Agriculture Systems, Chungnam National University, 99 Daehak-ro, Yuseong-gu, Daejeon 34134, Korea

## ⁯

***Dear Editor,***

Pinene (C_10_H_16_) is a well-known group of monoterpenes and the main component of turpentine, which is a fluid obtained by the distillation of resin harvested from coniferous trees, particularly those of the genus *Pinus* (Mercier et al., 2009[31]; Al-Tel et al., 2020[1]). Pinene can be divided into two structural isomers: α-pinene (α-pinene) and beta-pinene (β-pinene). α- and β-pinene are mainly produced by pine trees and many other conifers, as well as a wide range of herbs such as rosemary, parsley, basil, and even orange peel (Erman and Kane, 2008[9]; Vespermann et al., 2017[45]). 

α-pinene, the most abundant monoterpene in the atmosphere, accounts for more than 50 % of global monoterpene emissions and is a major component of phytoncides (Bagchi et al., 2020[2]; Li et al., 2009[27]). Phytoncides are antimicrobial allelochemical volatile organic compounds that are related to forest healing and activation of recreational forests. Trees are considered one of the major emitters of phytoncides (Li, 2010[26]).

A wide range of pharmacological activities of α- and β-pinene have been reported, such as anticoagulant, anti-inflammatory, anti-leishmania*,* antimalarial, antimicrobial, antioxidant, antitumor, analgesic, and antibiotic resistance modulation effects (Türkez and Aydın, 2016[42]; Salehi et al., 2019[40]). These monoterpenes exhibit various biological activities and have a wide range of applications, including development of antimicrobial and antiviral agents, flavors, fragrances, and fungicidal agents (Rivas da Silva et al., 2012[38]; Yang et al., 2013[49]). Herein, we summarize the recent published findings on the biological and pharmacological activities of pinene (Table 1(Tab. 1); References in Table 1: Bouzenna et al., 2017[3]; Cardoso et al., 2020[4]; Chen et al., 2015[5]; de Macêdo et al., 2018[6]; do Amaral et al., 2020[7]; Ensaka and Sakamoto, 2020[8]; Felipe et al., 2019[10]; Govindarajan et al., 2016[11]; Hou et al., 2019[12]; İnce et al., 2018[13]; Jensen et al., 2020[14]; Jo et al., 2021[15]; Karthikeyan et al., 2018[17], 2019[16]; Kasuya et al., 2015[18]; Khoshnazar et al., 2019[19], 2020[20]; Kim et al., 2015[21]; Kovač et al., 2015[22]; Kusuhara et al., 2019[23]; Langsi et al., 2020[24]; Lee et al., 2017[25]; Li et al., 2016[28]; Memariani et al., 2017[29]; Meng et al., 2020[30]; Min et al., 2020[32]; Moreira et al., 2016[33]; Nóbrega et al., 2020[34]; Pajaro-Castro et al., 2017[35]; Pinheiro et al., 2015[36]; Porres-Martínez et al., 2016[37]; Rodrigues et al., 2015[39]; Šimunović et al., 2020[41]; Ueno et al., 2019[44], 2020[43]; Wang et al., 2019[46]; Xu et al., 2018[47]; Yang et al., 2016[48]; Zamyad et al., 2019[50]; Zhang et al., 2015[52], 202[51]0; Zhao et al., 2018[53]).

EXCLI Journal 2021;20:812-818 – ISSN 1611-2156
Received: April 01, 2021, accepted: April 15, 2021, published: April 22, 2021

| **Key findings** | **Reference** |
|---|---|
| Natural killer (NK) cells are lymphocytes that can directly destroy cancer cells. α-pinene activates NK cells and increases NK cell cytotoxicity, suggesting that it is a potential compound for cancer immunotherapy. | Jo et al., 2021 |
| α-pinene has excellent antifungal activity, with high inhibitory activity against *Candida parapsilosis*. It has been proven to be effective in destroying fungal structures such as pseudohyphae and inducing a significant decrease in blastoconidia. | Nóbrega et al., 2020 |
| α-pinene and 3-carene prevent the development of immature stages of weevils and decrease the progeny to 94 %. These compounds are the most effective and promising novel phytoinsecticides for controlling maize weevils. | Langsi et al., 2020 |
| (+)-α-pinene combined with antimicrobial agents, such as amikacin, amoxicillin, cefepime, cefoxitin, and ceftazidime, showed a positive effect as it enhanced the antibiotic effect of these compounds. In addition, (+)-α-pinene induced cross-resistance only for the antimicrobial agents cefepime, ceftazidime, cefuroxime, and chloramphenicol. | do Amaral et al., 2020 |
| α-pinene treatment inhibits the increase in seizure incidences, whereas dizocilpine (MK-801) significantly reduces kindling acquisition. Administration of both these compounds inhibits pentylenetetrazole (PTZ)-induced activation of astrocytes and significantly inhibits the increase in extracellular matrix (ECM) molecules. These compounds might also prevent epileptic seizures by decreasing the activation of astrocytes and ECM molecule production. | Ueno et al., 2020 |
| α-pinene exerts neuroprotective effects during an ischemic stroke by reducing neuroinflammation and inhibiting apoptosis. Neuroinflammation and apoptosis play a vital role in neuronal death during ischemic stroke. | Khoshnazar et al., 2020 |
| α-pinene intake attenuates isoproterenol-induced inflammatory marker expression, thus significantly protecting against myocardial infarction and exerting cardioprotective and anti-inflammatory effects in male Wistar rats. | Zhang et al., 2020 |
| α-pinene odor stimulation is related to lipid metabolism, stress tolerance, and health span through specific signaling pathways. Thus, α-pinene odor might be a potential target for anti-aging and disease prevention. | Ensaka and Sakamoto, 2020 |
| (-)-α-pinene decreases quorum sensing in *Campylobacter*, which enhances the effects of antibiotics against various strains of *Campylobacter*, and the colonization of *Campylobacter* in broiler chickens. | Šimunović et al., 2020 |
| α-pinene and quercetin are bioactive phytocompounds present in the soil. They may affect the tolerance of microorganisms to persistent toxicants and change their impact on the environment. | Cardoso et al., 2020 |
| *Bursaphelenchus xylophilus* is a parasitic nematode that causes pine wilt disease, which results in severe damage to pine trees worldwide. α-pinene protects the host species during the early *B. xylophilus* infection and colonization stages. | Meng et al., 2020 |
| α-pinene enhances osteoblast differentiation, which was confirmed by alkaline phosphatase and alizarin red S staining. This effect might be due to the attenuation of tumor necrosis factor-alpha (TNFα) induction by α-pinene, which leads to the inhibition of Smad1/5/9 phosphorylation and extracellular matrix mineralization. | Min et al., 2020 |
| α-pinene has significant toxic effects on *Folsomia candida*. In addition, α-pinene might improve their cold stress tolerance significantly at membrane concentrations of more than 87 mmol $kg^{-1}$. | Jensen et al., 2020 |
| α-pinene showed neuroprotective effect in an ischemic stroke rat model by restoring antioxidant enzymatic activity, attenuating lipid peroxidation, and reducing inflammation in the ischemic brains. | Khoshnazar et al., 2019 |

| Key findings | Reference |
|---|---|
| α-pinene showed potential anticancer effects in PA-1 cancer cells via induction of cytotoxicity and inhibition of cell sequence development along with programmed cell death. | Hou et al., 2019 |
| α-pinene reduces MK-801-induced behavioral abnormalities, which are similar to those observed in neuropsychiatric disorders. Based on this finding, it can be suggested that plants and oils rich in α-pinene might have a potential effect on schizophrenia treatment. | Ueno et al., 2019 |
| α-pinene shows low toxicity to pinewood nematode for a short period of time. It affects the diversity and abundance of the symbiotic bacteria com-munity of these nematodes, which might have a potential impact on the de-velopment and reproduction of nematodes. | Wang et al., 2019 |
| A fragrant environment (FE) containing α-pinene activated the immune system and hypothalamus/sympathetic nerve/leptin axis in mice, leading to the retardation of growth of tumor cells, which was confirmed by the neuro-hormonal and immunological changes. | Kusuhara et al., 2019 |
| α-pinene is the major constituent of *Ducrosia anethifolia* essential oil, and it can induce anticonvulsant and antioxidant effects in rats. | Zamyad et al., 2019 |
| α-pinene treatment effectively prevents UVA-induced lipid peroxidation in mouse skin probably through its antioxidant property. | Karthikeyan et al., 2019 |
| α-pinene did not show anticonvulsant properties in PTZ-induced male Swiss albino mice. This might be attributed to the structure of the compound, because pretreatment with β-pinene decreased the intensity of seizures and prolonged the death time of PTZ-treated albino mice. In addition, α-pinene reduced the concentration of norepinephrine, dopamine, and nitrite but not of thiobarbituric acid reactive substance during PTZ-induced seizure. | Felipe et al., 2019 |
| α-pinene inhibits *miR221* gene expression, leading to G2/M-phase cell cycle arrest of hepatocellular carcinoma cells (HepG2), and triggers the ATM-p53-Chk2 and CDKN1B/p27-CDK1 pathway, which leads to the suppression of hepatoma tumor development in humans. Thus, α-pinene could be considered a highly potential chemotherapeutic agent for hepatocellular carcinoma treatment. | Xu et al., 2018 |
| β-pinene exerts potential antifungal activity by weakening the cell wall through interactions with Delta-14-sterol reductase and 1,3-β-glucan synthase. These molecules could effectively decrease the adhesion of *Candida* biofilm. | de Macêdo et al., 2018 |
| α-pinene prevents oxidative stress caused by ultraviolet A (UVA) exposure, inflammation, UVA-induced DNA damages, and apoptosis in human skin epidermal keratinocytes. | Karthikeyan et al., 2018 |
| α-pinene and cineole are the main components present in *Rosmarinus officinalis* (RO) that contributed to the increased survival of flap necrosis. This finding shows that RO has anti-inflammatory effects. | İnce et al., 2018 |
| α-pinene prevented the growth of human prostate cancer cells, induced programmed cell death, and arrested the cell cycle in the PC-3 cell line. In addition, α-pinene-treated mice inhibited tumor progression more effectively than control mice. α-pinene inhibited the growth of prostate cancer cells in a xenograft model. Thus, it might be an effective therapeutic agent for the treatment of prostate cancer. | Zhao et al., 2018 |
| α-pinene treatment significantly increased scopolamine-induced cognitive dysfunction. In addition, it efficiently reduced the mean escape latency in Morris water-maze test. Mainly, α-pinene treatment increases choline acetyltransferase levels in the cortex and protein levels of antioxidant enzymes (heme oxygenase-1 and manganese superoxide dismutase) in the hippocampus by triggering the nuclear factor erythroid 2-related factor 2 (NRF2). These findings suggest that α-pinene could be a potential neuroprotective compound for the management of dementia-related learning and memory loss. | Lee et al., 2017 |

| Key findings | Reference |
|---|---|
| β-pinene and linalool act as repellents against *Tribolium castaneum*. β-pinene showed potential effect compared with linalool in stimulating insect repellency. A significant change in the expression of genes associated with neuronal transmission was observed. | Pajaro-Castro et al., 2017 |
| α-pinene has potential antioxidant capacity. Exposure of cells to α-pinene together with aspirin showed a significant increase in cell survival and GSH level. However, malondialdehyde (MDA), total super dismutase (SOD), and Mn-SOD activity were decreased. This might be due to the activation of p38 mitogen-activated protein kinases (MAPKs) and inhibition of c-Jun N-terminal kinase (JNK) by α-pinene. These results suggest that α-pinene can protect IEC-6 cells against aspirin-induced oxidative stress. | Bouzenna et al., 2017 |
| α-pinene is present in the oil extract of *Pistacia atlantica*. It has a protective effect against ethanol-induced gastric ulcer and antibacterial activity against *Helicobacter pylori*. | Memariani et al., 2017 |
| (−)-α-pinene has various beneficial effects such as anxiolytic, antioxidant, and anti-inflammatory properties. In mice, (−)-α-pinene improved non-rapid eye movement sleep (NREMS) without disturbing NREMS intensity by delaying gamma-aminobutyric acid (GABA)ergic synaptic transmission, which acts as a partial modulator of GABAA-benzodiazepine (BZD) receptors and directly binds to the distinct BZD binding sites of GABAA receptor. Thus, (−)-α-pinene might be used as an effective therapeutic agent against anxiety and sleeping disorders. | Yang et al., 2016 |
| α-pinene and 1,8-cineole are monoterpenes that showed effective antioxidant activity and protected rat pheochromocytoma (PC12) cells from $H_2O_2$-induced oxidative stress. This study showed the potential therapeutic benefits of these compounds in maintaining antioxidant balance in nervous system diseases. | Porres-Martínez et al., 2016 |
| (-)-β-pinene (βP)/β-cyclodextrin (β-CD) complex shows an antihypertensive effect, which has been proven in pharmacological studies. βP induces endothelium-independent vasorelaxation by decreasing $Ca^{2+}$ influx through L-type $Ca^{2+}$ channel associated with a decrease in calcium sensitivity. | Moreira et al., 2016 |
| α-pinene, linalool, and 1-octanol are the active ingredients present in frankincense oil extracts. Topical application of this oil showed significant analgesic and anti-inflammatory effects via the inhibition of cyclooxygenase-2 overexpression and nociceptive stimulus-induced inflammatory infiltrates. | Li et al., 2016 |
| α-pinene from the oil extract of *Plectranthus barbatus* was found to be an effective mosquito control agent. It is effective even at low doses and can be used as an alternative safe compound for controlling mosquitoes. | Govindarajan et al., 2016 |
| (-)-α-pinene can modulate antibiotic resistance in *Campylobacter jejuni* via various mechanisms such as metabolic disruption, decreased membrane integrity, and microbial efflux inhibition. Based on this finding, it can be suggested that (-)-α-pinene can be used to control *Campylobacter* antibiotic-resistant strains in future. | Kovač et al., 2015 |
| α-pinene extracted from pine needle oil exhibits anti-cancer activity. It inhibited the growth of liver cancer BEL-7402 cells with an inhibitory rate of 79.3 % and 69.1 % in *in vitro* and *in vivo* experiments, respectively. In addition, the levels of Chk1 and Chk2 were upregulated, whereas CDK1, CDC25, and Cyclin B levels were downregulated. | Chen et al., 2015 |
| α-pinene exhibits anti-inflammatory activity through the inhibition of the nuclear factor-kappa B and mitogen-activated protein kinase pathway in mouse peritoneal macrophages. This finding shows that α-pinene has an anti-inflammatory effect, and it can be used as a potential candidate for the treatment of various inflammatory diseases. | Kim et al., 2015 |
| α-pinene shows significant antiulcerogenic activity. A good correlation between α-pinene concentration and gastroprotective activity of *Hyptis* species was also reported. | Pinheiro et al., 2015 |

| Key findings | Reference |
|---|---|
| α-pinene is the major constituent in *Syzygium cumini* essential oil; it exerts significant anti-leishmania activity by modulating macrophage activation, with suitable levels of cytotoxicity in murine macrophages and human red blood cells. | Rodrigues et al., 2015 |
| α-pinene concentration increase in the brain leads to an increase in locomotor activity as well as the expression level of tyrosine hydroxylase mRNA. These anxiolytic-like effects might be associated with both neurological and pharmacological transfer. | Kasuya et al., 2015 |
| Non-small-cell lung cancer (NSCLC) cells treated with α-pinene and β-pinene combined with paclitaxel (PAL), i.e., (α-pinene+PAL) and (β-pinene+PAL), showed changes in morphological characteristics; apoptosis-like condensation of chromatin and breakdown of the nucleus were observed. | Zhang et al., 2015 |

## *Conflict of interest*

The authors declare no conflict of interest.

## Acknowledgements

This work was supported by the National Research Foundation of Korea (NRF) grant funded by the Korea government (MSIT) (No. 2021R1A2C201017811) and this study was carried out with the support of ‘R&D Program for Forest Science Technology (Project No. 2021379B10-2123-BD02)’ provided by Korea Forest Service (Korea Forestry Promotion Institute).

## Conflict of interest

The authors declare no conflict of interest.

## Figures and Tables

**Table 1:**
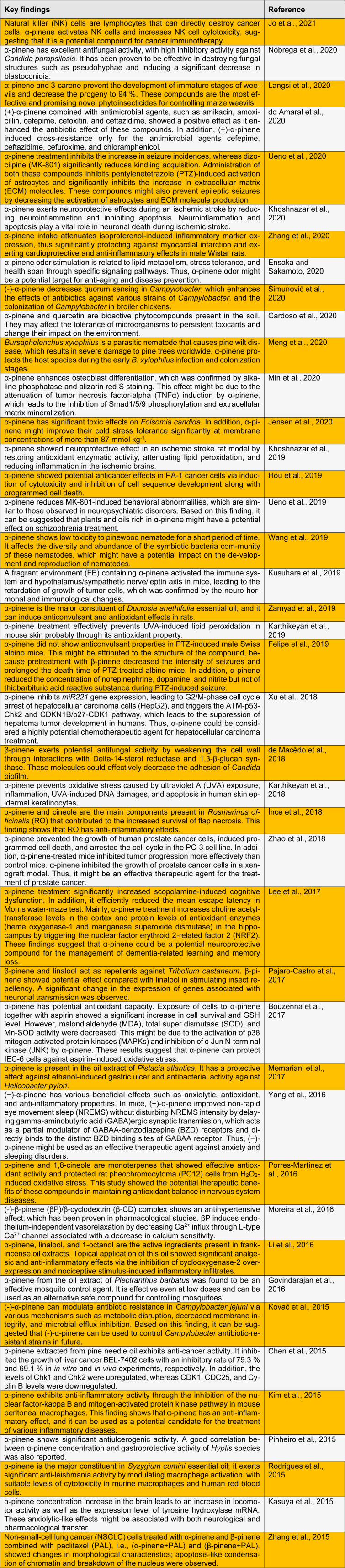
Recent studies on the biological and pharmacological activities of pinene

## References

[1] Al-Tel TH, Tarazi H, Aloum LO, Lorke DE, Petroianu GA (2020). Possible metabolic conversion of pinene to ionone. Pharmazie.

[2] Bagchi A, Yu Y, Huang JH, Tsai CC, Hu WP, Wang CC (2020). Evidence and evolution of Criegee intermediates, hydroperoxides and secondary organic aerosols formed via ozonolysis of α-pinene. Phys Chem Chem Phys.

[3] Bouzenna H, Hfaiedh N, Giroux-Metges MA, Elfeki A, Talarmin H (2017). Potential protective effects of alpha-pinene against cytotoxicity caused by aspirin in the IEC-6 cells. Biomed Pharmacother.

[4] Cardoso P, Nunes T, Pinto R, Sá C, Matos D, Figueira E (2020). Rhizobium response to sole and combined exposure to cadmium and the phytocompounds alpha-pinene and quercetin. Ecotoxicology.

[5] Chen W, Liu Y, Li M, Mao J, Zhang L, Huang R (2015). Anti-tumor effect of α-pinene on human hepatoma cell lines through inducing G2/M cell cycle arrest. J Pharmacol Sci.

[6] de Macêdo Andrade AC, Rosalen PL, Freires IA, Scotti L, Scotti MT, Aquino SG, de Castro RD (2018). Antifungal activity, mode of action, docking prediction and anti-biofilm effects of (+)-β-pinene enantiomers against Candida spp. Curr Top Med Chem.

[7] do Amaral FLE, Farias TC, de Brito RC, de Melo TR, Ferreira PB, Lima ZN, e al (2020). Effect of the association and evaluation of the induction to adaptation of the (+)-α-pinene with commercial antimicrobials against strains of Escherichia coli. Curr Top Med Chem.

[8] Ensaka N, Sakamoto K (2020). α-Pinene odor exposure enhances heat stress tolerance through Daf-16 in Caenorhabditis elegans. Biochem Biophys Res Commun.

[9] Erman MB, Kane BJ (2008). Chemistry around pinene and pinane: a facile synthesis of cyclobutanes and oxatricyclo-derivative of pinane from cis- and trans-pinanols. Chem Biodivers.

[10] Felipe CFB, Albuquerque AMS, de Pontes JLX, de Melo JÍV, Rodrigues TCML, de Sousa AMP (2019). Comparative study of alpha- and beta-pinene effect on PTZ-induced convulsions in mice. Fundam Clin Pharmacol.

[11] Govindarajan M, Rajeswary M, Hoti SL, Bhattacharyya A, Benelli G (2016). Eugenol, α-pinene and β-caryophyllene from Plectranthus barbatus essential oil as eco-friendly larvicides against malaria, dengue and Japanese encephalitis mosquito vectors. Parasitol Res.

[12] Hou J, Zhang Y, Zhu Y, Zhou B, Ren C, Liang S (2019). α-Pinene induces apoptotic cell death via caspase activation in human ovarian cancer cells. Med Sci Monit.

[13] İnce B, Dadacı M, Kılınç İ, Oltulu P, Yarar S, Uyar M (2018). Effect of cineole, alpha-pinene, and camphor on survivability of skin flaps. Turk J Med Sci.

[14] Jensen TG, Holmstrup M, Madsen RB, Glasius M, Trac LN, Mayer P (2020). Effects of α-pinene on life history traits and stress tolerance in the springtail Folsomia candida. Comp Biochem Physiol C Toxicol Pharmacol.

[15] Jo H, Cha B, Kim H, Brito S, Kwak BM, Kim ST (2021). α-pinene enhances the anticancer activity of natural killer cells via ERK/AKT pathway. Int J Mol Sci.

[16] Karthikeyan R, Kanimozhi G, Madahavan NR, Agilan B, Ganesan M, Prasad NR (2019). Alpha-pinene attenuates UVA-induced photoaging through inhibition of matrix metalloproteinases expression in mouse skin. Life Sci.

[17] Karthikeyan R, Kanimozhi G, Prasad NR, Agilan B, Ganesan M, Srithar G (2018). Alpha pinene modulates UVA-induced oxidative stress, DNA damage and apoptosis in human skin epidermal keratinocytes. Life Sci.

[18] Kasuya H, Okada N, Kubohara M, Satou T, Masuo Y, Koike K (2015). Expression of BDNF and TH mRNA in the brain following inhaled administration of α-pinene. Phytother Res.

[19] Khoshnazar M, Bigdeli MR, Parvardeh S, Pouriran R (2019). Attenuating effect of α-pinene on neurobehavioural deficit, oxidative damage and inflammatory response following focal ischaemic stroke in rat. J Pharm Pharmacol.

[20] Khoshnazar M, Parvardeh S, Bigdeli MR (2020). Alpha-pinene exerts neuroprotective effects via anti-inflammatory and anti-apoptotic mechanisms in a rat model of focal cerebral ischemia-reperfusion. J Stroke Cerebrovasc Dis.

[21] Kim DS, Lee HJ, Jeon YD, Han YH, Kee JY, Kim HJ (2015). Alpha-pinene exhibits anti-inflammatory activity through the suppression of MAPKs and the NF-κB pathway in mouse peritoneal macrophages. Am J Chin Med.

[22] Kovač J, Šimunović K, Wu Z, Klančnik A, Bucar F, Zhang Q (2015). Antibiotic resistance modulation and modes of action of (-)-α-pinene in Campylobacter jejuni. PLoS One.

[23] Kusuhara M, Maruyama K, Ishii H, Masuda Y, Sakurai K, Tamai E (2019). A fragrant environment containing α-pinene suppresses tumor growth in mice by modulating the hypothalamus/sympathetic nerve/leptin axis and immune system. Integr Cancer Ther.

[24] Langsi JD, Nukenine EN, Oumarou KM, Moktar H, Fokunang CN, Mbata GN (2020). Evaluation of the insecticidal activities of α-pinene and 3-carene on Sitophilus zeamais motschulsky (Coleoptera: Curculionidae). Insects.

[25] Lee GY, Lee C, Park GH, Jang JH (2017). Amelioration of scopolamine-induced learning and memory impairment by α-pinene in C57BL/6 mice. Evid Based Complement Alternat Med.

[26] Li Q (2010). Effect of forest bathing trips on human immune function. Environ Health Prev Med.

[27] Li Q, Kobayashi M, Wakayama Y, Inagaki H, Katsumata M, Hirata Y (2009). Effect of phytoncide from trees on human natural killer cell function. Int J Immunopathol Pharmacol.

[28] Li XJ, Yang YJ, Li YS, Zhang WK, Tang HB (2016). α-Pinene, linalool, and 1-octanol contribute to the topical anti-inflammatory and analgesic activities of frankincense by inhibiting COX-2. J Ethnopharmacol.

[29] Memariani Z, Sharifzadeh M, Bozorgi M, Hajimahmoodi M, Farzaei MH, Gholami M (2017). Protective effect of essential oil of Pistacia atlantica Desf. on peptic ulcer: Role of α-pinene. J Tradit Chin Med.

[30] Meng F, Li Y, Liu Z, Wang X, Feng Y, Zhang W (2020). Potential molecular mimicry proteins responsive to α-pinene in Bursaphelenchus xylophilus. Int J Mol Sci.

[31] Mercier B, Prost J, Prost M (2009). The essential oil of turpentine and its major volatile fraction (alpha- and beta-pinenes): A review. Int J Occup Med Environ Health.

[32] Min HY, Son HE, Jang WG (2020). Alpha-pinene promotes osteoblast differentiation and attenuates TNFα-induced inhibition of differentiation in MC3T3-E1 pre-osteoblasts. Clin Exp Pharmacol Physiol.

[33] Moreira IJ, Menezes PP, Serafini MR, Araújo AA, Quintans-Júnior LJ, Bonjardim LR (2016). Characterization and antihypertensive effect of the complex of (-)-β- pinene in β-cyclodextrin. Curr Pharm Biotechnol.

[34] Nóbrega JR, Silva DF, Andrade Júnior FP, Sousa PMS, Figueiredo PTR, Cordeiro LV (2020). Antifungal action of α-pinene against Candida spp. isolated from patients with otomycosis and effects of its association with boric acid. Nat Prod Res.

[35] Pajaro-Castro N, Caballero-Gallardo K, Olivero-Verbel J (2017). Neurotoxic effects of linalool and β-pinene on Tribolium castaneum Herbst. Molecules.

[36] Pinheiro MDA, Magalhães RM, Torres DM, Cavalcante RC, Mota FS, Oliveira Coelho EM (2015). Gastroprotective effect of alpha-pinene and its correlation with antiulcerogenic activity of essential oils obtained from Hyptis species. Pharmacogn Mag.

[37] Porres-Martínez M, González-Burgos E, Carretero ME, Gómez-Serranillos MP (2016). In vitro neuroprotective potential of the monoterpenes α-pinene and 1,8-cineole against H2O2-induced oxidative stress in PC12 cells. Z Naturforsch C J Biosci.

[38] Rivas da Silva AC, Lopes PM, Barros de Azevedo MM, Costa DC, Alviano CS, Alviano DS (2012). Biological activities of α-pinene and β-pinene enantiomers. Molecules.

[39] Rodrigues KA, Amorim LV, Dias CN, Moraes DF, Carneiro SM, Carvalho FA (2015). Syzygium cumini (L.) Skeels essential oil and its major constituent α-pinene exhibit anti-Leishmania activity through immunomodulation in vitro. J Ethnopharmacol.

[40] Salehi B, Upadhyay S, Erdogan Orhan I, Kumar Jugran A, Jayaweera SLD, Dias DA (2019). Therapeutic potential of α- and β-pinene: A miracle gift of nature. Biomolecules.

[41] Šimunović K, Sahin O, Kovač J, Shen Z, Klančnik A, Zhang Q (2020). (-)-α-Pinene reduces quorum sensing and Campylobacter jejuni colonization in broiler chickens. PLoS One.

[42] Türkez H, Aydın E (2016). In vitro assessment of cytogenetic and oxidative effects of α-pinene. Toxicol Ind Health.

[43] Ueno H, Shimada A, Suemitsu S, Murakami S, Kitamura N, Wani K (2020). Alpha-pinene and dizocilpine (MK-801) attenuate kindling development and astrocytosis in an experimental mouse model of epilepsy. IBRO Rep.

[44] Ueno H, Shimada A, Suemitsu S, Murakami S, Kitamura N, Wani K (2019). Attenuation effects of alpha-pinene inhalation on mice with dizocilpine-induced psychiatric-like behaviour. Evid Based Complement Alternat Med.

[45] Vespermann KA, Paulino BN, Barcelos MC, Pessôa MG, Pastore GM, Molina G (2017). Biotransformation of α- and β-pinene into flavor compounds. Appl Microbiol Biotechnol.

[46] Wang X, Yu Y, Ge J, Xie B, Zhu S, Cheng X (2019). Effects of α-pinene on the pinewood nematode (Bursaphelenchus xylophilus) and its symbiotic bacteria. PLoS One.

[47] Xu Q, Li M, Yang M, Yang J, Xie J, Lu X (2018). α-pinene regulates miR-221 and induces G2/M phase cell cycle arrest in human hepatocellular carcinoma cells. Biosci Rep.

[48] Yang H, Woo J, Pae AN, Um MY, Cho NC, Park KD (2016). α-pinene, a major constituent of pine tree oils, enhances non-rapid eye movement sleep in mice through GABAA-benzodiazepine receptors. Mol Pharmacol.

[49] Yang J, Nie Q, Ren M, Feng H, Jiang X, Zheng Y (2013). Metabolic engineering of Escherichia coli for the biosynthesis of alpha-pinene. Biotechnol Biofuels.

[50] Zamyad M, Abbasnejad M, Esmaeili-Mahani S, Mostafavi A, Sheibani V (2019). The anticonvulsant effects of Ducrosia anethifolia (Boiss) essential oil are produced by its main component alpha-pinene in rats. Arq Neuropsiquiatr.

[51] Zhang B, Wang H, Yang Z, Cao M, Wang K, Wang G (2020). Protective effect of alpha-pinene against isoproterenol-induced myocardial infarction through NF-κB signaling pathway. Hum Exp Toxicol.

[52] Zhang Z, Guo S, Liu X, Gao X (2015). Synergistic antitumor effect of α-pinene and β-pinene with paclitaxel against non-small-cell lung carcinoma (NSCLC). Drug Res (Stuttg).

[53] Zhao Y, Chen R, Wang Y, Yang Y (2018). α-pinene inhibits human prostate cancer growth in a mouse xenograft model. Chemotherapy.

